# Emerging of Anomalous Higher‐Order Topological Phases in Altermagnet/Topological Insulator Heterostructure by Floquet Engineering

**DOI:** 10.1002/advs.202522203

**Published:** 2026-01-31

**Authors:** Donghao Wang, Arnob Kumar Ghosh, Yongchun Tao, Fusheng Ma, Cheng Song

**Affiliations:** ^1^ Department of Physics Nanjing Normal University Nanjing Jiangsu China; ^2^ Department of Physics and Astronomy Uppsala University Uppsala Sweden; ^3^ Key Laboratory of Advanced Materials (MOE) School of Materials Science and Engineering Tsinghua University Beijing China

**Keywords:** altermagnet, floquet theory, higher‐order topological phases, topological insulator

## Abstract

Altermagnetism, characterized by collinear‐compensated magnetic ordering with vanishing net magnetization, has opened new frontiers in topological matter through its distinctive spin‐momentum locking mechanism. We demonstrate that the altermagnetism in altermagnet/topological insulator (TI) heterostructure breaks time‐reversal symmetry, and in combination with Floquet engineering, gives rise to anomalous higher‐order topological insulator (AHOTI) phases with pure/hybridized π‐corner modes (CMs). Two fundamental breakthroughs by Floquet engineering are revealed. First, three distinct topological phase transitions are established: trivial‐to‐AHOTI with pure π‐CMs (AHOTI‐I), higher‐order TI‐to‐AHOTI with hybridized π‐CMs (AHOTI‐II), and mutual transitions between AHOTI‐I and ‐II. Second, the real‐space locations of topological CMs—not only conventional 0‐ but also anomalous π‐CMs—are synchronously manipulated by Néel vector reorientation, establishing an altermagnetism‐programmable paradigm for topological state localization. The emergent topological phases are systematically characterized through the nested dynamical Wilson loop formalism, deliberately adapted for Floquet‐driven systems, which shows exact correspondence with the observed CMs. This work establishes a universally versatile platform for non‐equilibrium dynamical control of topological phase, thereby providing transformative implications for reconfigurable topological electronics and fault‐tolerant quantum computing architectures.

## Graphical abstract

We combine altermagnet with topological insulators and subject the structure to Floquet driving. This breaks time‐reversal symmetry and creates a new type of higher‐order topological insulator. Its key feature is the emergence of programmable “0/*π*‐corner modes” that can be controlled by magnetic field direction, offering a novel dynamic platform for topological electronics.

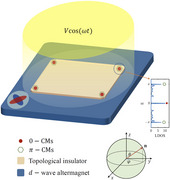

## 1 Introduction

Over decades, the exploration of topological phases has formed a cornerstone of condensed matter physics. Conventional topological insulators (TIs) with time‐reversal symmetry (TRS) are characterized by their gapless edge states, which adheres to the principle of bulk‐edge correspondence [1, 2, 3, 4, 5, 6]. Recent developments have extended this framework to higher‐order topological insulators (HOTIs) [7, 8, 9, 10, 11, 12], which are characterized by the emergence of (d−n)‐dimensional protected edge states, with d the dimension of the system and n (2≤n≤d) the order of the topological phase. For example, a 2D HOTI hosts zero‐dimensional corner modes (CMs) [13], while a three‐dimensional second‐order TI supports one‐dimensional hinge states [14]. Notably, the concept of HOTIs has transitioned from theoretical predictions to experimental observations, with paradigmatic examples including bismuth [15], ${\mathrm{Bi}}_{4}{\mathrm{Br}}_{4}$ [16, 17], and ${\mathrm{WTe}}_{2}$ [18, 19]. Beyond these crystalline materials, the experimental realization of HOTI has been successfully extended to diverse artificial platforms encompassing photonic crystals [20], sonic crystals [21, 22], and synthetic electric‐circuit networks [23], as comprehensively reviewed in Ref. [24]. While HOTIs have been extensively studied, research on their anomalous counterparts—anomalous higher‐order topological insulators (AHOTIs)—remains scarce. AHOTIs originating from Floquet engineering applied to static systems, where periodic driving is employed to manipulate band topology and give rise to π‐CMs. Unlike static HOTIs, AHOTIs challenge the conventional bulk‐boundary correspondence by hosting robust π‐CMs that have no static analogue. These dynamical modes have been proposed as auxiliary degrees of freedom to facilitate the topologically protected exchange of Majorana zero modes within a single quantum wire [25]. Moreover, such π modes are the promising candidates for implementing fundamental operations in topological quantum computation within Floquet systems [26, 27]. It is demonstrated that utilizing time vortices can bind topologically protected π Majorana modes at space‐time defects [28]. Therefore, the AHOTIs manifest themselves as a critical frontier in non‐equilibrium topological matter by the π‐CMs' distinctive properties.[Supplementary-material advs74048-supl-0001]

Altermagnetism, a recently discovered magnetism, merges collinear antiferromagnetic spin order with momentum‐dependent spin splitting akin to ferromagnets—yet with zero net magnetization [29, 30, 31]. Unlike conventional magnets, altermagnets (AMs) exhibit non‐relativistic spin polarization in reciprocal space, breaking TRS and Kramers degeneracy. Their band structures feature spin‐polarized “islands” with d−,g−, or i‐wave symmetry in momentum space, as observed in materials like ${\mathrm{RuO}}_{2}$ [32, 33, 34, 35, 36], ${\mathrm{MnF}}_{2}$ [37, 38], MnTe [39, 40, 41], CrSb [42, 43] and in engineered structures such as twisted magnetic van der Waals bilayers [44]. These traits give rise to phenomena such as the anomalous Hall effect [38, 40, 41, 45, 46], Nernst effect [47, 48], and finite momentum Cooper pairs in AM‐superconductor junctions [49]. In particular, the strong momentum‐dependent spin splitting provides a powerful knob to manipulate spin‐dependent electronic structures and break symmetries essential for inducing novel topological phases, such as higher‐order topology when interfaced with TIs [50, 51]. The Néel vector orientation in AMs acts as an additional controllable degree of freedom, allowing for selective activation and spatial manipulation of CMs [52].

**FIGURE 1:**
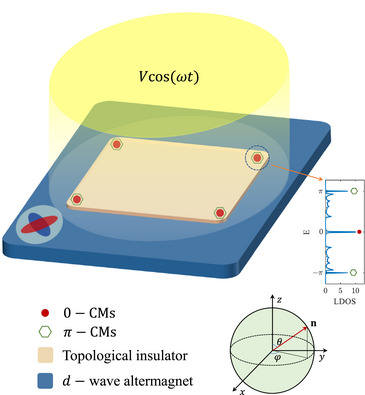
The schematic diagram of the proposed AM/TI heterostructure with the proximity‐induced d‐wave altermagnetism in the TI. An external Floquet driving is directly imposed on the TI, thereby realizing AHOTI phase with pure/hybridized π‐CMs. The right‐side inset displays the LDOS of the system, obtained by projecting the density of states for a driving period T=1 onto the corner regions. The sharp peaks observed at energies E=0 and ±π correspond to the 0‐ and π‐CMs, respectively, demonstrating the coexistence of both types of modes.

In this article, by synergizing the unique symmetry‐breaking properties of altermagnetism with Floquet engineering—a powerful theoretical framework for non‐equilibrium topological systems, we demonstrate the realization of AHOTIs and unveil their underlying topological phase transition mechanisms. An AM/TI heterostructure (Figure 1), subjected to Floquet engineering protocols, is constructed. The increase of the driving period T (i.e., ω decreases) can turn the trivial phase into an AHOTI with pure π‐CMs (AHOTI‐I) and drive the HOTI into an AHOTI with the hybridization of 0 and π‐CMs (AHOTI‐II) in spatial distribution. Furthermore, tuning the Bernevig, Hughes, and Zhang (BHZ) mass term $m_{0}$ [53] directly regulates edge‐state to enable the interconversion between AHOTI‐I and ‐II phases. Crucially, the Néel vector orientation determines CMs' spatial distribution—the out‐of‐plane vector makes the CMs locate at the four corners, while the in‐plane one confines them in the diagonal corners tunable via the azimuthal angle. The various topological phases, validated by not only the dynamic nested Wilson loop theory but also the gap opening in the 2D Floquet TI edge state, correspond perfectly with the presence of CMs. Such synergy between altermagnetism and Floquet driving opens avenues for designing reconfigurable topological circuits and fault‐tolerant quantum memories.

## 2 Results

### 2.1 Model Hamiltonian and Floquet Engineering of Topological Phase Transitions

To investigate the interplay of altermagnetism and Floquet engineering in generating higher‐order topology, we employ the BHZ model on a square lattice. This model is particularly suited for our purpose as it provides a paradigmatic, minimal description of the quantum spin Hall insulator phase, with well‐understood topological invariants [1, 53] and experimental evidence [54]. Crucially, its square‐lattice symmetry naturally accommodates the d‐wave form factor of the proximitized altermagnet, thereby preserving the $C_{4}$ crystalline symmetry essential for protecting and manipulating higher‐order corner modes. The BHZ model's extensive use in the study of both static and Floquet‐driven topological phases further facilitates the interpretation of our results within an established theoretical framework. While other lattice geometries such as the hexagonal Kane‐Mele model also realize $Z_{2}$ topological phases, the square‐lattice BHZ model offers the most direct and symmetry‐compatible setting for exploring the synergistic effects of d‐wave altermagnetic coupling and periodic driving.

Within this framework, we consider a higher‐order topological system consisting of a TI proximitized to a d‐wave AM, as depicted in Figure 1. The static Hamiltonian can be written as

(1) $$\begin{array}{cc} H(k)=M(k) & \Gamma_{1}+A_{x}\sin k_{x}\Gamma_{2}+A_{y}\sin k_{y}\Gamma_{3}\\ & +J\left(k\right)s\cdot n⊗\sigma_{x} \end{array}$$

with $\Gamma_{1}=s_{0}⊗\sigma_{z},\Gamma_{2}=s_{y}⊗\sigma_{x}$, and $\Gamma_{3}=s_{x}⊗\sigma_{x}$, where the Pauli matrices $s_{i}$ and $\sigma_{i}\left(i\in x,y,z\right)$ correspond to the spin (↑↓) and orbital (a,b) degrees of freedom, respectively, and $s_{0}$ denotes the 2×2 identity matrix. In Equation (1), the first three terms describe a conventional BHZ TI with a non‐trivial $Z_{2}$ invariant, with $M\left(k\right)=\left(m_{0}-t_{x}\cos k_{x}-t_{y}\cos k_{y}\right)$. In the last term, $J\left(k\right)=J\left(\cos k_{x}-\cos k_{y}\right)$ with J denoting the strength of the altermagnetism, encapsulates the essential d‐wave symmetry and spin‐momentum locking of the proximitized AM. Furthermore, the vector n=(sinθcosφ,sinθsinφ,cosθ) given by the last term, represents the pivotal Néel vector orientation, a unique and controllable degree of freedom intrinsic to AM, with θ the polar angle and φ the azimuthal angle in spherical coordinates. The Néel vector orientation is set with θ=φ=0 for the out‐of‐plane scenario and θ=π/2 for the in‐plane one.

The advent of Floquet theory has revolutionized the study of non‐equilibrium topological matter. Subjecting topological systems to a periodic driving can dynamically manipulate their topological properties [55, 56]. For instance, Floquet driving can induce transitions from trivial to topological phases, even generating exotic π‐modes that defy static system symmetries [57, 58, 59, 60]. These features not only expand the catalog of achievable topological phases but also enable real‐time control over edge states, offering pathways toward fault‐tolerant quantum devices. Recent work highlights how Floquet protocols can engineer anomalous edge responses, such as chiral edge currents in driven graphene [61, 62] or time‐crystalline surface states in topological superconductors (TSC) [63, 64].

We incorporate Floquet driving into the on‐site mass term of static Hamiltonian. The driving scheme is constructed as

(2) $$V\left(t\right)=V_{F}\cos\left(\omega t\right)\Gamma_{1}$$

with $V_{F}$ the driving amplitude and ω=2π/T the driving frequency. Then, the complete dynamic Hamiltonian is given by H(k,t)=H(k)+V(t), which is evidently periodic, i.e., H(k,t)=H(k,t+T). The approaches for obtaining the Floquet quasi‐energy band structure and local density of states (LDOS), are described in detail in the Methods section.

**FIGURE 2:**
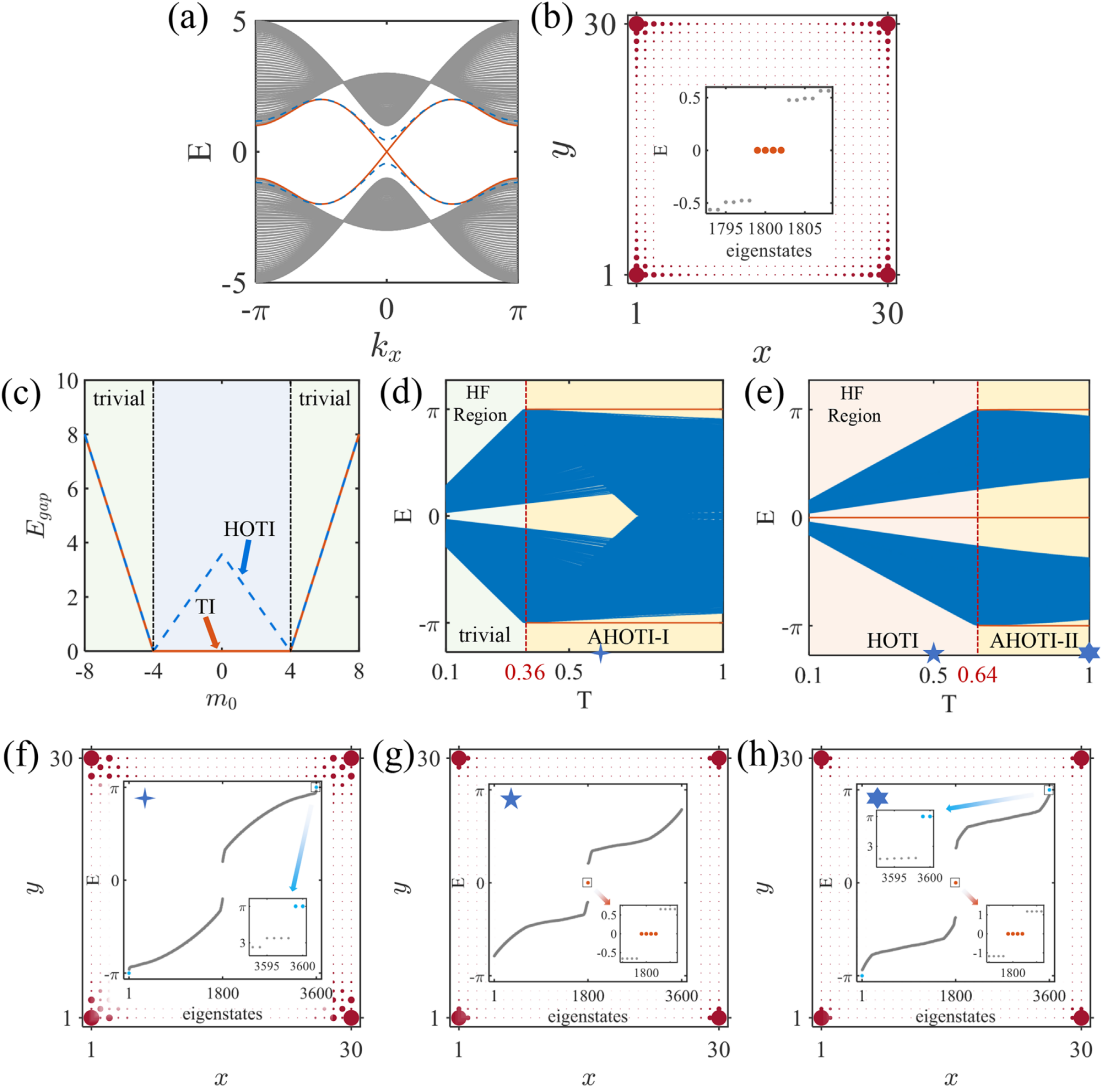
Evolution of topological states and phase transitions in the out‐of‐plane scenario. (a) Helical edge states of the AM/TI heterostructure for J=0 (red solid line) and J=1 (blue dashed line). (b) Spectrum diagram and LDOS plot of CMs for the system with AM under OBC. (c) Edge state gap versus $m_{0}$ and the corresponding phase. For J=0 (solid curves), the system hosts a conventional TI phase in the central region ($-4<m_{0}<4$) with trivial phases elsewhere, and for J=1 (dashed curves), a topological phase transition to HOTI in the same parameter range with the triviality preserved outside this interval. Floquet driving band evolution and topological transitions under 2D OBC: (d) with initial trivial phase for $m_{0}=5$ and (e) with initial HOTI phase for $m_{0}=1$. The red solid lines represent the 0‐ and π‐CMs. Energy spectrum and LDOS of CMs (J=1): (f) Corresponding to (d) with T=0.6, (g) and (h) corresponding to (e) with T=0.5 and T=1, respectively.

For the static regime in the out‐of‐plane scenario, the helical edge states of the BHZ model exhibit distinct behaviors depending on J. At J=0, the system retains gapless helical edge states under cylindrical boundary conditions (see the red solid line in Figure 2a). However, introducing a finite J=1 opens a gap in these edge states (see the blue dashed line in Figure 2a), accompanied by the emergence of 0‐CMs under OBC (Figure 2b), confirming the transition to a HOTI. The topological phase diagram is further identified in Figure 2c, where the edge state gap Δ evolves with $m_{0}$. The critical points at $m_{0}=\pm \left(t_{x}+t_{y}\right)$ — derived from band closure conditions at $\left(k_{x},k_{y}\right)=\left(0,0\right)$ and (π,π) — separate the trivial phase regime ($|m_{0}|>t_{x}+t_{y}$) from the topological one ($|m_{0}|<t_{x}+t_{y}$). The derivation of phase transition points and the expanded topological phase diagram in the ($m_{0},J$) parameter space are provided in the Supporting Information [65].

Upon introducing Floquet driving, the equilibrium topology is dynamically reconfigured, enabling two T‐dependent transitions. First, starting from the trivial phase ($|m_{0}|>t_{x}+t_{y}$, consistent with Figure 2c), increasing the driving period T induces a transition to an AHOTI‐I phase, as shown in Figure 2d. The AHOTI‐I phase hosts π‐CMs at quasienergy π/T—a hallmark of Floquet engineering absent in equilibrium systems. Second, beginning from the static HOTI phase ($|m_{0}|<t_{x}+t_{y}$, gapped regime in Figure 2c), increasing T triggers a reentrant transition to an AHOTI‐II phase (Figure 2e). In the phase, hybridized CMs that the original 0‐CMs (from altermagnetism) coexist with Floquet‐induced π‐CM, are supported.

The introduction of Floquet driving induces topological phase transitions in the system that transcend those of static frameworks, giving rise to AHOTI‐I and ‐II phases. To elucidate the regulatory mechanisms by which driving period T governs the topological evolution, we first analyze the continuous variation of the driving period T in the high‐frequency (HF) region. When the driving frequency ω significantly exceeds the bandwidth of the static Hamiltonian (i.e., T→0), the system's behavior is predominantly governed by the zeroth‐order term $h_{\omega }^{\left(0\right)}$ of the Floquet Hamiltonian, whose topological properties align exactly with those of the static system. This characteristic is directly verified in Figure 2d. Specifically, at T<0.36, the system resides in a trivial phase, exhibiting neither 0‐ nor π‐CMs, which matches exactly the trivial phase shown in Figure 2c.

As the driving period T increases, the system gradually deviates from the HF regime, exhibiting unique topological responses governed by Floquet engineering. Notably, when T reaches 0.36 (Figure 2d), π‐CMs emerge in the energy spectrum (see Figure 2f), indicating a topological phase transition from the trivial phase to the AHOTI‐I phase.

For the system initially in the HOTI phase (corresponding to the HF regime in Figure 2e), the modulation of driving period T reveals richer phase transition pathways. At T=0.5 (Figure 2g), the system retains HOTI characteristics, with spatial distributions of 0‐CMs closely mirroring those of the static system (Figure 2b). However, when T increases to 1.0 (Figure 2h), the system exhibits coexistence of 0‐CMs and π‐CMs, unveiling a HOTI → AHOTI‐II phase transition. The coexistence of 0‐ and π‐CMs is a hallmark of Floquet systems. The 0‐CMs are protected by the topology of the static Hamiltonian, which is inherited and modified by the time‐averaged component of the Floquet drive, and reside in the quasi‐energy gap around zero. In contrast, the π‐CMs are purely non‐equilibrium phenomena, generated by the periodic driving itself, and are protected by the topology associated with the π‐gap in the Floquet quasi‐energy spectrum. These two gaps are distinct and separable in the frequency domain, allowing both types of topologically robust modes to coexist simultaneously without conflict. This dual‐energy CMs coexistence serves as direct evidence for the existence of Floquet‐induced AHOTI‐II phases, originating from the synergy between the static altermagnetic order and the dynamic Floquet modulation.

Importantly, since the distinction between the initial trivial phase and HOTI phase lies solely in the BHZ mass term $m_{0}$, the AHOTI‐I and ‐II phases generated via Floquet modulation can mutually transform within overlapping driving period ranges by tuning $m_{0}$. Further details are provided in the Supporting Information [65].

**FIGURE 3:**
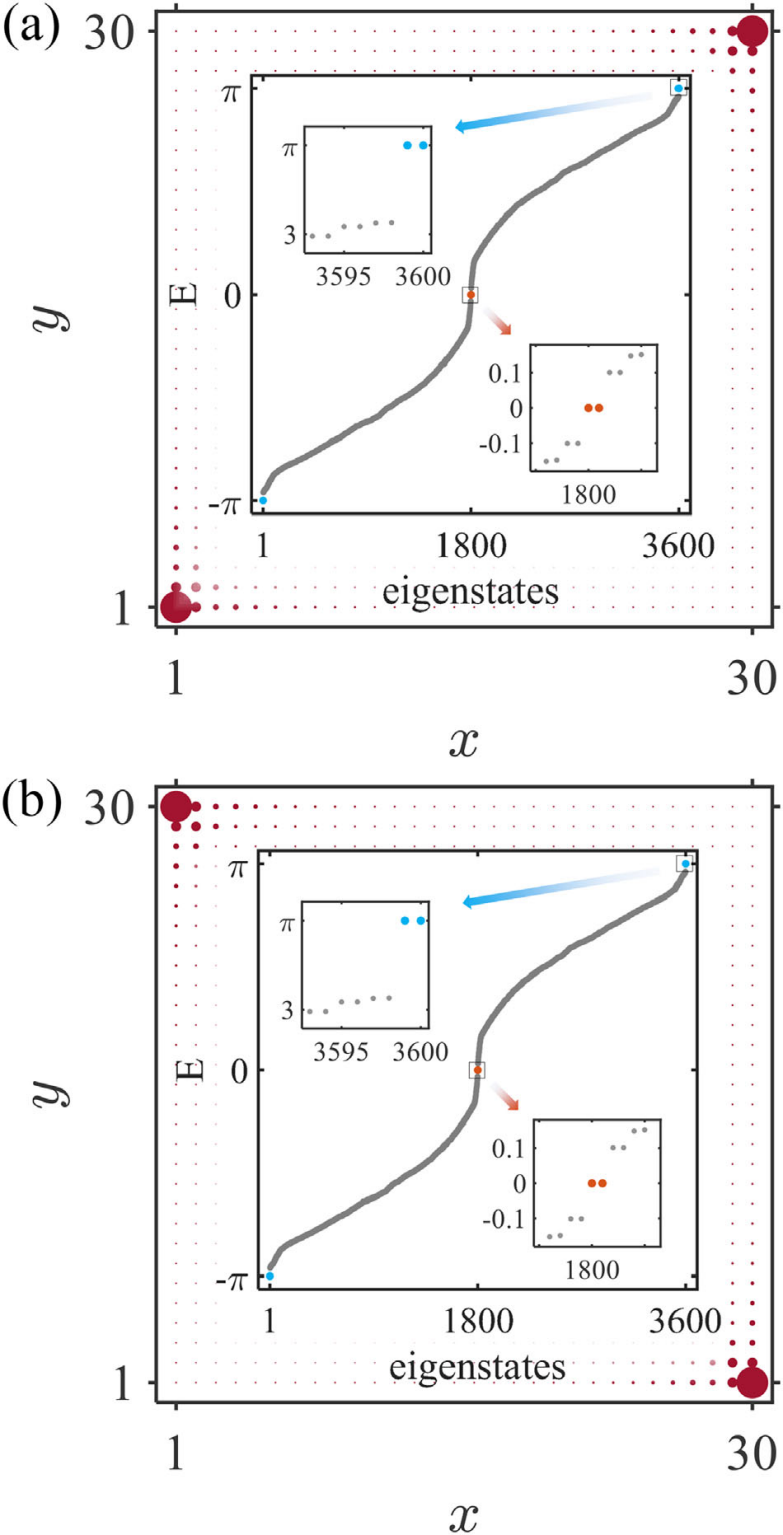
The in‐plane scenario with θ=π/2. Energy spectrum and LDOS of CMs for the azimuthal angles (a) φ=7π/4 and (b) π/4 with T=1.2,J=0.5, and $m_{0}=1$.

It is particularly noteworthy that the regulation of CMs' spatial distributions by the Néel vector orientation exhibits pronounced anisotropic characteristics. When the Néel vector aligns along the out‐of‐plane direction (Figure 2f–h), the CMs display homogeneous spatial distributions. However, when the vector rotates into the in‐plane orientation (Figure 3), the localization properties of CMs manifest strong azimuthal φ‐dependence. At φ=7π/4 and φ=π/4 (corresponding to Néel vectors along (11) and $\left(1\bar{1}\right)$ directions, respectively), both 0‐ and π‐CMs exclusively localize in diagonal regions of the sample, with their distribution directions strictly collinear with the Néel vector. This phenomenon arises from the breaking of $C_{4}$ rotational symmetry induced by in‐plane anisotropy of the AM, directly controlled by the Néel vector orientation.

### 2.2 Dynamic Topological Characterization of CMs

The topological phase transitions induced by the driving period T are far richer than the aforementioned basic transitions. Our detailed calculations reveal that upon increasing T, the system undergoes a series of topological phase transitions, exhibiting multiple switches between the trivial, HOTI, AHOTI‐I, and AHOTI‐II phases (see Supporting Information [65] for detials). To complement this characterization, we compute the real‐space bulk quadrupole moment Q as a static topological invariant [66, 67, 68, 69]. A value of Q=0.5 serves as a definitive signature of a non‐trivial topological phase hosting either pure 0‐ or π‐CMs. However, this invariant exhibits significant limitations in Floquet systems. Q cannot distinguish whether the topological state resides in the 0‐ or π‐gap, as it lacks energy resolution. Furthermore, it reduces to Q=0 in the AHOTI‐II phase where both 0‐ and π‐CMs coexist, thus coinciding with the value for the trivial phase. The specific formula for Q and the corresponding numerical simulation results are provided in Supporting Information [65].

These limitations of static invariants motivate the necessity for an energy‐resolved dynamical characterization capable of uniquely identifying both 0‐ and π‐CMs. We therefore employ the dynamical nested Wilson loop method [58, 59], which provides an unambiguous topological characterization of the 0‐ and π‐CMs through the time evolution of the average dynamical polarization $\left\langle \nu_{y,\mu^{\prime }}^{\left({\pm \nu }_{x}\right)}\right\rangle \left(t\right)$ (see Methods for details). This approach not only overcomes the shortcomings of the quadrupole moment but also demonstrates the powerful capability and rich physics of Floquet engineering in manipulating higher‐order topological states.

**FIGURE 4:**
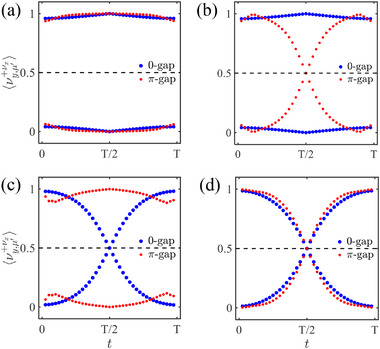
The out‐of‐plane scenario with J=1. (a)–(d) The average dynamical polarization for trivial, AHOTI‐I, HOTI, and AHOTI‐II phases, respectively, with the corresponding $\left(m_{0},T\right)$ being [(5,0.2), (5,0.6), (1,0.5), (1,1)].

The plots of $\left\langle \nu_{y,\mu^{\prime }}^{\left({\pm \nu }_{x}\right)}\right\rangle \left(t\right)$ for four varied cases are illustrated in Figure 4a–d, where (a) and (b) correspond to the trivial and AHOTI‐I phases in Figure 2d, respectively, while (c) and (d) are the counterparts of the HOTI and AHOTI‐I ones in Figure 2e, respectively. In the topologically trivial regime, the time‐dependent average polarization $\left\langle \nu_{y,\mu^{\prime }}^{\left({\pm \nu }_{x}\right)}\right\rangle \left(t\right)$ for both 0‐ and π‐gaps demonstrates periodicity restoration — initial values (0 or 1) at t=0 fully recover at t=T [Figure 4a]. This periodic restoration breaks down in higher‐order topological phases due to CMs' generation. For the static HOTI phase containing only 0‐CMs, the zero‐gap polarization undergoes complete inversion (0→1) across the temporal cycle [Figure 4b], with intersecting spectral branches signaling the presence of 0‐CMs. Analogous inversion is shared by the AHOTI‐I phase with exclusive π‐CMs [Figure 4c], establishing a one‐to‐one correspondence between π‐CMs and π‐gap spectral crossings. For AHOTI‐II phase (coexisting 0‐ and π‐CMs), dual‐gap polarization inversion occurs simultaneously [Figure 4d], where both 0‐ and π‐gap branches develop typical intersections, providing an explicit signature for hybrid topology. The absence of branch crossings in trivial phases reflects unrestricted cyclic evolution. In contrast, the emergence of intersections in topological phases stems from particle transfer between adjacent unit cells. Open boundaries geometrically restrict this motion, preventing full polarization restoration and causing corner‐localization. This dynamical distinction enables unambiguous identification of 0‐ and π‐CMs through respective $\left\langle \nu_{y,\mu^{\prime }}^{\left({\pm \nu }_{x}\right)}\right\rangle \left(t\right)$ branchs crossing patterns.

### 2.3 Quasi‐Energy Spectrum of the Floquet Hamiltonian

**FIGURE 5:**
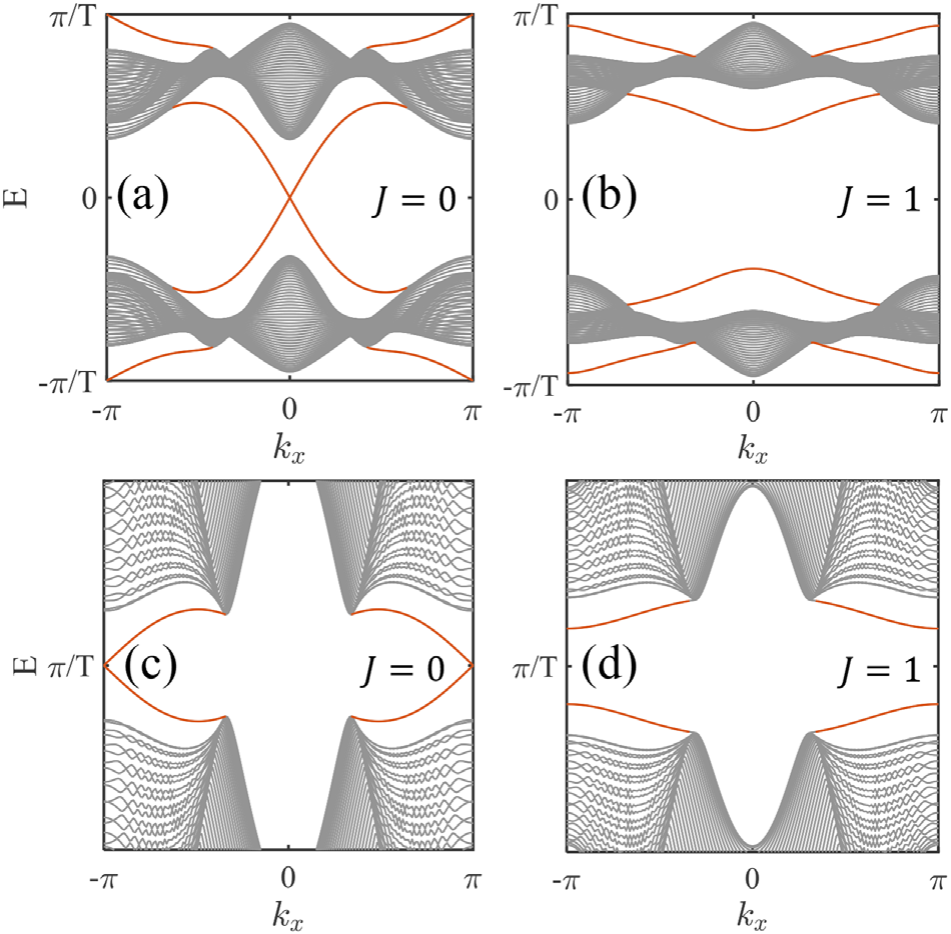
Edge states of the system under quasi‐1D boundary conditions for the out‐of‐plane scenario, which are highlighted in red solid lines. Here, $m_{0}=1,T=1$ for (a) and (b); $m_{0}=5,T=0.5$ for (c) and (d).

In this section, we gain further insight into the HOTI or AHOTI phase by studying the Floquet Hamiltonian's quasienergy spectrum. The quasienergy spectra of Floquet Hamiltonians exhibit frequency periodicity, specifically, the range E∈[−π/T,π/T] can be analogized to the “energy spectrum Brillouin zone” of $H_{F}$, with spectral replicas outside this range [60, 70]. When truncating the infinite‐dimensional $H_{F}$ matrix for numerical calculations, spectral distortions predominantly occur at the zone boundaries, while the features within [−π/T,π/T] remain intact. We identify two characteristic Floquet phases, one is the anomalous first‐order TI phase (AFOTI‐I) featuring solely first‐order π‐edge states (see Figure 5c), the other is the hybrid anomalous first‐order TI phase (AFOTI‐II) hosting coexisting 0‐ and π‐edge states (as shown in Figure 5a). However, due to the breaking of TRS induced by altermagnetism, the energy gaps for the edge states being previously closed around π and/or 0 are opened, companied by the emergence of π and/or 0‐CMs. For instance, Figure 5b,d correspond to Figure 2(h) and (g) respectively, where the edge states exhibit a gap after the introduction of altermagnetism. The corresponding π and/or 0‐CMs, suggesting the phase transition from AFOTI phases to AHOTI ones, highlights the interplay between Floquet engineering and altermagnetism. In the Supplement [65], we comprehensively summarize all topological phases discussed in the main text and their phase transition pathways.

## 3 Discussion

To summarize, we construct an AM/TI heterostructure leveraging the unique properties of altermagnetism combined with Floquet engineering. This system exhibits three distinct topological phase transitions in both out‐of‐plane and in‐plane altermagnetism scenarios: (i) Trivial‐to‐AHOTI‐I transition induced by increasing the driving period T; (ii) HOTI‐to‐AHOTI‐II transition under T‐enhancement; (iii) AHOTI‐I and ‐II interconversion governed by the BHZ model's mass term $m_{0}$. Crucially, the CMs are distributed in the four corners for the out‐of‐plane scenario, while the in‐plane altermagnetism localizes them in the diagonal corners with positions tunable via the Néel vector's azimuthal angle. The resulting 0 and π‐CMs are identified by the average dynamic polarization $\left\langle \nu_{y,\mu^{\prime }}^{\left({\pm \nu }_{x}\right)}\right\rangle \left(t\right)$ using the dynamic nested Wilson loop method. Their presence is signaled by the intersection of the two $\left\langle \nu_{y,\mu^{\prime }}^{\left({\pm \nu }_{x}\right)}\right\rangle \left(t\right)$ branches within the corresponding quasienergy gaps. The absence of branch crossings in trivial phases reflects unrestricted cyclic evolution of the Wannier centers. In contrast, the emergence of intersections in topological phases stems from particle transfer between adjacent unit cells being obstructed by the presence of topologically protected, corner‐localized states. This dynamical distinction establishes a direct bulk‐corner correspondence, enabling unambiguous identification of 0‐ and π‐CMs through the respective $\left\langle \nu_{y,\mu^{\prime }}^{\left({\pm \nu }_{x}\right)}\right\rangle \left(t\right)$ branch crossing patterns. Moreover, topological phase transitions, such as from AFOTI to AHOTI phases, are further confirmed by the opening of quasienergy gaps in the Floquet spectrum. It is worth pointing out that the topological nature of the AHOTI phases ensures an inherent robustness against weak static disorder. The CMs, being localized and pinned in the quasienergy gaps, are protected as long as the disorder potential does not close the bulk gap and destroy the underlying topological phase.

These findings demonstrate that, therefore, altermagnetism provides an indispensable and versatile platform due to its compensated order and Néel vector tunability. Synergized with Floquet driving, it enables novel pathways for manipulating higher‐order topological phases and 0/π‐CMs, affording new strategies for topological quantum computing and information processing.

## 4 Material Considerations and Experimental Feasibility

The practical experimental feasibility of our proposal is high and rests on several well‐established pillars. There are currently multiple experimental candidates for TI materials, such as ${\left(\mathrm{Bi},\mathrm{Sb}\right)}_{2}{\mathrm{Te}}_{3}$ [71, 72, 73] and HgTe/CdTe [74]. As for altermagnetism, it has been observed in various materials, including metallic ones like ${\mathrm{RuO}}_{2}$ [32, 33], CrSb [42, 43], and ${\mathrm{Mn}}_{5}{\mathrm{Si}}_{3}$ [46, 75], and insulating ones like ${\mathrm{MnF}}_{2}$ [37, 38] and MnTe [39]. Furthermore, theoretical studies have extensively explored various approaches to implement Floquet driving in material systems. For example, elliptically polarized light irradiation has been verified as an effective method [76], and alternative schemes utilizing THz oscillating magnetic fields or in‐plane electric fields have been proposed for realizing Floquet TIs in HgTe‐based structures [77]. Therefore, our proposed AM/TI heterostructure represents an experimentally feasible platform that can be investigated using established spectroscopic techniques. Experimental detection protocols and realistic parameter estimates for experimental validation of the predicted topological phases are systematically outlined in the Supplement [65].

Beyond these general material and driving considerations, we address several specific questions concerning the symmetry choice of the altermagnet, the experimental viability of the heterostructure, and the robustness of our predictions under realistic perturbations—all of which further strengthen the experimental relevance of our work.

*Symmetry choice of the altermagnet*: Our focus on d‐wave altermagnetism is motivated by its prominence in established material candidates (e.g., ${\mathrm{RuO}}_{2}$ and ${\mathrm{MnF}}_{2}$) and its theoretical clarity as a fundamental symmetry channel for spin‐split antiferromagnets [29, 30, 32, 37]. The d‐wave symmetry naturally couples to the square‐lattice BHZ model, preserving the $C_{4}$ crystalline symmetry essential for protecting higher‐order corner modes. While altermagnets with higher even‐parity symmetries (e.g., g‐wave or i‐wave) could lead to distinct spatial patterns of corner modes, recent theoretical work has shown that altermagnets with different even‐parity symmetries share a common physical origin and can be described within a unified minimal model [78]. This implies that the core mechanism of Floquet‐induced higher‐order topology is qualitatively robust across symmetry classes, with quantitative variations in mode distribution and phase boundaries. Furthermore, strain‐induced transitions between symmetry classes (e.g., g‐wave to d‐wave) have been predicted in materials like ${\mathrm{FeS}}_{2}$ and ${\mathrm{Nb}}_{2}{\mathrm{FeB}}_{2}$ [79], opening interesting future directions for exploring tunable topological responses. Nonetheless, the d‐wave case remains the most suitable starting point at present: its material realization is the most clearly established, its theoretical model is the most transparent, and it provides an indispensable conceptual foundation for studying the synergy between altermagnetic order and Floquet driving.

*Lattice mismatch and proximity effect*: The construction of high‐quality TI/AM heterostructures faces two practical challenges, lattice mismatch and the penetration of the magnetic proximity effect. Both can be addressed with existing material synthesis techniques. Lattice mismatch can be effectively mitigated by van der Waals epitaxy [80], which relies on weak interlayer forces and has enabled the growth of high‐quality interfaces such as ${\mathrm{Bi}}_{2}{\mathrm{Se}}_{3}$/NiFe and ${\mathrm{Bi}}_{2}{\mathrm{Se}}_{3}$/${\mathrm{La}}_{0.7}{\mathrm{Sr}}_{0.3}{\mathrm{MnO}}_{3}$ [81, 82]. In these systems, efficient spin transport and strong interfacial magnetic coupling have been demonstrated experimentally. To ensure the proximity effect influences the entire TI layer, ultrathin TI films (a few nanometers thick) are routinely used. At this scale, the interfacial magnetic proximity effect is sufficient to dominate the electronic states of the TI. These advances validate the heterostructure platform assumed in our model.

*Role of spin–orbit coupling (SOC)*: The role of SOC in altermagnetism touches upon a core aspect of understanding the physical nature of this emerging magnetic order and its controllability. The defining non‐degenerate spin splitting arises from non‐relativistic exchange interactions and crystal symmetry, not from SOC [31]. This purity of physical origin is key to altermagnetism being recognized as a distinct magnetic order and ensures the stability of its core electronic structure features across many candidate materials. However, SOC is essential for the dynamic control of the Néel vector, for example through spin–orbit torques. In our theoretical treatment, the Néel vector orientation is taken as a tunable parameter to isolate its symmetry‐breaking effect on topology which is a reasonable and common practice at this stage of theoretical research.

*Robustness against interfacial Rashba spin–orbit coupling (RSOC)*: The breaking of inversion symmetry at the TI/AM interface is likely to induce RSOC. Nevertheless, experimental studies on TI/magnetic‐insulator heterostructures show that topological surface states remain clearly resolved and dominate the system's response even in the presence of such interfacial effects [83]. Theoretical work further indicates that RSOC can coexist with topological phases and may even enhance certain spin‐dependent responses through constructive interference [84]. Other studies also lend supporting evidence to the view that interfacial effects often act as tunable parameters [85, 86]. Therefore, we expect the main predictions of our work to be qualitatively robust against moderate RSOC's perturbations. The RSOC effect is more likely to play the role of fine‐tuning the system's parameters, for instance, causing shifts in the width of quasi‐energy gaps, the precise energy of CMs, or the critical driving strength for phase transitions. Such quantitative adjustments do not affect the fundamental conclusion regarding the existence of topologically protected, position‐tunable Floquet CMs.

Furthermore, we expect that our theoretical framework extends naturally to TSC systems, especially higher‐order TSCs [87, 88, 89, 90, 91, 92, 93, 94, 95, 96, 97, 98] and their anomalous counterparts. The relevant higher‐order topological phases are predicted to host Majorana 0‐ and π‐CMs, whose non‐Abelian statistics make them prime candidates for robust, fault‐tolerant topological quantum computation. The ability to create, manipulate, and interconvert between these exotic phases using the combined knobs of altermagnetism and periodic driving opens a new frontier in the pursuit of dynamical quantum control and topologically protected quantum information processing.

Here, it is pointed out that, from first‐principles calculations, investigating the topological properties of altermagnet/TI heterostructures can be a promising future direction. Such calculations would allow us to quantify the proximity‐induced coupling and interface effects, as well as to examine the influence of factors like RSOC and lattice mismatch on the system's electronic structure [52, 79].

## 5 Methods

### 5.1 Floquet Driving Protocol

Although our study employs a cosine driving protocol via mass‐term modulation (corresponding to elliptically polarized light), other driving schemes—such as circularly polarized light or more complex periodic modulations—are also expected to generate the AHOTI phases [57, 76]. This is because the key mechanism relies on the general principles of Floquet engineering combined with altermagnetic symmetry breaking, and is not sensitive to the specific form of the drive. In the following, we outline the general approach based on Floquet–Bloch theory. The Floquet Hamiltonian matrix elements within the extended Hilbert space can be written as,

(3) $${\left(H_{F}\right)}_{nm}=h_{\omega }^{n-m}+\omega n\delta_{nm},\;n,m\in Z$$

Specifically, $h_{\omega }^{n-m}=\frac{1}{T}\int_{0}^{T}H\left(k,t\right)e^{i(n-m)\omega t}dt$, where the terms

(4) $$h_{\omega }^{\left(0\right)}=H\left(k\right)\left(\cos\left(\omega t\right)\to 0\right)$$

(5) $$h_{\omega }^{\left(1\right)}=\frac{V_{F}}{2}\left(s_{0}⊗\sigma_{z}\right)$$

with $h_{\omega }^{\left(-1\right)}={\left(h_{\omega }^{\left(1\right)}\right)}^{†}$, can be readily computed. The notation “→0” here signifies that the time‐dependent perturbation V(t) is averaged out over one full driving period when constructing the zeroth‐order component $h_{\omega }^{\left(0\right)}$ of the Floquet Hamiltonian. Specifically, $h_{\omega }^{\left(0\right)}$ is given by $\frac{1}{T}\int_{0}^{T}H\left(k,t\right)dt$. The integral of the static part H(k) over a period trivially yields itself. In contrast, the integral of a periodically oscillating term, such as $V_{F}\cos\left(\omega t\right)\Gamma_{1}$, averages to zero over its complete cycle. Consequently, for zeroth order, the time‐dependent drive is averaged out, and $h_{\omega }^{\left(0\right)}$ effectively reduces to the undriven static Hamiltonian H(k). This treatment is foundational to high‐frequency Floquet perturbation theory and holds rigorously in the high‐frequency limit where the drive frequency ω is much larger than the bandwidth of the static system.

Due to the infinite‐dimensional characteristic of the Floquet Hamiltonian $H_{F}$, we are confronted with computational challenges, and thus have to resort to the time evolution operator, otherwise known as the Floquet operator [57, 99, 100]

(6) $$\begin{array}{cc} U(k,t) & =\mathrm{TO}\exp\left[-i\int_{0}^{t}H\left(k,t\right)dt\right]\\ & =\lim_{\Delta t\to 0}\prod_{n=1}^{N}e^{-iH(k,t-n\Delta t)\Delta t} \end{array}$$

with TO denoting the time‐ordering operator. By setting the time t in U(k,t) equal to the period T, we can determine the dynamic Hamiltonian's evolution over a complete driving cycle. The eigenenergy spectrum and the LDOS of the CMs for the system under 2D open boundary condition (OBC) are obtained by utilizing U(k,T). All theoretical calculations in this work are performed with the following parameter settings. The BHZ model parameters are fixed at $A_{x}=A_{y}=t_{x}=t_{y}=2$. The Floquet driving amplitude $V_{F}=2$ and the 2D OBC is constructed on a 30×30 lattice grid.

### 5.2 Characterization Methods

Utilizing the time evolution operator, we establish the “micromotion operator” capable of characterizing the normal and anomalous dynamics within Floquet systems,

(7) $$U_{\varepsilon }\left(k,t\right)=U\left(k,t\right){\left[U\left(k,T\right)\right]}_{\varepsilon }^{-\frac{t}{T}}$$

with ε denoting the branch cut of the exponential function. Here, $U_{\varepsilon }\left(k,t\right)$ maintains periodic invariance, i.e., $U_{\varepsilon }\left(k,t\right)=U_{\varepsilon }\left(k,t+T\right)$, particularly, for different branch cut, the results given by $U_{\varepsilon }\left(k,t\right)$ are identical at t=T.

Based on the formalism of $U_{\varepsilon }\left(k,t\right)$, we can compute the average dynamical polarization of CMs using the dynamical nested Wilson loop theory,

(8) $$\left\langle \nu_{y,\mu^{\prime }}^{\left({\pm \nu }_{x}\right)}\right\rangle \left(t\right)=\frac{1}{L_{x}}\sum_{k_{x}}\nu_{y,\mu^{\prime }}^{\left(\pm \nu_{x}\right)}\left(k_{x},t\right)$$

where $\nu_{y,\mu^{\prime }}^{\left(\pm \nu_{x}\right)}\left(k_{x},t\right)$ are obtained from the diagonalization of the second‐order dynamical Wilson loop operator. Full analytical derivations of the “micromotion operator” and the averaged dynamical polarization are detailed in the Supporting Information [65].

## Author Contributions

D.W. developed the theoretical model, performed the numerical calculations, and wrote the initial draft of the manuscript. A.K.G. contributed to the development and implementation of the nested dynamical Wilson loop formalism and provided significant theoretical guidance. Y.T., F.M., and C.S. supervised the project, conceived the key ideas, and were responsible for manuscript organization, and finalization. All the authors reviewed the manuscript.

## Conflicts of Interest

The authors declare no conflicts of interest.

## Supporting information


**Supporting File**: advs74048‐sup‐0001‐SuppMat.pdf.

## Data Availability

Data underlying the results presented in this paper are not publicly available at this time but may be obtained from the authors upon reasonable request.
