# Supplementary material for: Emerging of Anomalous Higher‐Order Topological Phases in Altermagnet/Topological Insulator Heterostructure by Floquet Engineering
Source: Adv Sci (Weinh). 2026 Jan 31;13(19):e22203. doi: 10.1002/advs.202522203 (PMC13045236; doi:10.1002/advs.202522203)
Supplement: Supplementary file 1 — Supporting File: advs74048‐sup‐0001‐SuppMat.pdf. [file ADVS-13-e22203-s001.pdf]

Supplemental Materials for "Emerging of Anomalous Higher-Order Topological Phases in Altermagnet/Topological Insulator Heterostructure by Floquet Engineering"

Donghao Wang[1], Arnob Kumar Ghosh[2], Yongchun Tao[1,*], Fusheng Ma[1,*], and Cheng Song[3,*]
[1]Department of Physics, Nanjing Normal University, Nanjing 210023, Jiangsu, China
[2]Department of Physics and Astronomy, Uppsala University, Box 516, 75120 Uppsala, Sweden
[3]Key Laboratory of Advanced Materials (MOE), School of Materials Science and Engineering, Tsinghua University, Beijing, China
(Dated: January 4, 2026)

Contents

S-1. Effects of Altermagnetism on Topological Phase Transitions in the BHZ Model 1

S-2. Floquet-Driven Modulation Pathways of Topological Phase Transitions 2

S-3. Floquet Micromotion Operators and Dynamical Nested Wilson Loop Theory 3
A. Construction of Floquet Micromotion Operators 3
B. Dynamical Nested Wilson Loop Theory 3

S-4. Topological Phase Characterization via Real-Space Bulk Quadrupole Moment 4

S-5. Experimental Detection Scheme and Parameter Estimation 5
A. Experimental Detection of Topological State and Altermagnetism 5
B. Experimental Parameters Estimation and Realization Pathways 6

References 6

S-1. EFFECTS OF ALTERMAGNETISM ON TOPOLOGICAL PHASE TRANSITIONS IN THE BHZ MODEL

The introduction of out-of-plane altermagnetism breaks the time-reversal symmetry of the system, thereby triggering the topological phase transition. By investigating the bulk band closure conditions in the AM/TI (BHZ model), we establish a theoretical framework for the static topological phase diagram.

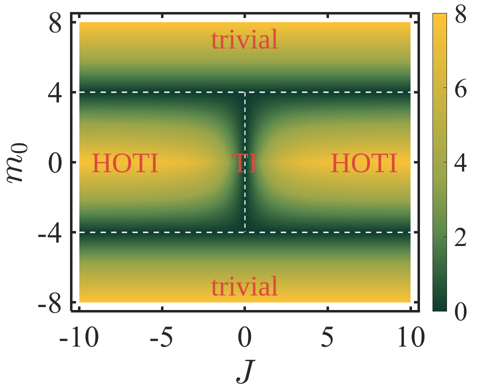

Figure S1: Topological phase diagram of the static AM/TI model in parameter space (m_0, J), where the color palette shows the band gap E_{gap}. The results shown in Figure 2(c) of the main text are corresponding to $J = 0$ and $J = 1$ of this diagram.

---

*E-mail: yctao88@163.com
*E-mail: phymafs@njnu.edu.cn
*E-mail: songcheng@mail.tsinghua.edu.cn

For the AM/TI model with out-of-plane Néel vector, its static Hamiltonian in momentum space adopts a 4×4 block matrix form

$$H(\mathbf{k}) = \begin{bmatrix} M(\mathbf{k})\sigma_z + J(\cos k_x - \cos k_y)\sigma_x & A_y \sin k_y\sigma_x - iA_x \sin k_x\sigma_y \\ A_y \sin k_y\sigma_x + iA_x \sin k_x\sigma_y & M(\mathbf{k})\sigma_z - J(\cos k_x - \cos k_y)\sigma_x \end{bmatrix}, \tag{S1}$$

where the mass term $M(\mathbf{k}) = m_0 - t_x \cos k_x - t_y \cos k_y$, and σ_i denote Pauli matrices. Analytical diagonalization yields the doubly degenerate energy spectrum

$$E_{\mathbf{k}} = \pm\frac{1}{\sqrt{2}}\left[M(\mathbf{k})^2 + A_x^2 \sin^2 k_x + A_y^2 \sin^2 k_y + J^2(\cos k_x - \cos k_y)^2\right]^{1/2}. \tag{S2}$$

Notably, the altermagnetic term $J(\cos k_x - \cos k_y)$ vanishes automatically at the high-symmetry points $\Gamma(0, 0)$ and $\mathrm{M}(\pi, \pi)$ in the Brillouin zone. Consequently, the bandgap closure condition reduces to $M(\mathbf{k}) = 0$, leading to the critical values of mass parameter,

$$m_0^{\mathrm{crit}} = \pm(t_x + t_y). \tag{S3}$$

This indicates that the introduction of out-of-plane altermagnetism can not change m_0^{crit} for bandgap closure. However, through calculations of the edge-state gap in one-dimensional boundaries and the LDOS of CMs [see Figure 2(b) in the main text], we reveal that altermagnetism alters the topological classification, i.e., the original nontrivial first-order TI phase evolves into HOTI phase characterized by CMs. The phase diagram presented in Figure S1 clearly illustrates the topological phase distribution modulated by both m_0 and J, where the color map quantifies the edge-state gap magnitude under quasi-one-dimensional boundary conditions, providing direct visualization of topological distinctions across parameter regimes for different phases.

S-2. FLOQUET-DRIVEN MODULATION PATHWAYS OF TOPOLOGICAL PHASE TRANSITIONS

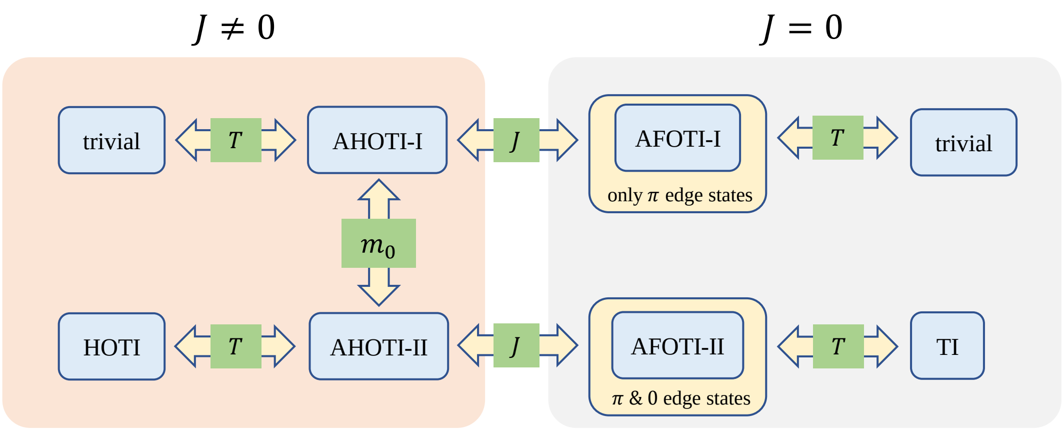

Figure S2: Flowchart of topological phase transitions subjected to synergetic effects of the altermagnetism and Floquet driving.

Figure S2 depicts the topological phase transition processes under the synergetic effects of altermagnetism and Floquet driving, which we specifically describe below, including the involved multistage phase transition mechanisms and dynamic modulation principles. (a) Combination of the altermagnetism and Floquet driving ($J \neq 0$ regime): The initial trivial phase evolves into the AHOTI-I phase hosting pure π-CMs, the original HOTI phase transforms into the AHOTI-II phase featuring the coexistence of 0- and π-CMs, and mutual transitions between the AHOTI-I and AHOTI-II phases are achieved by tuning the mass term m_0. (b) In the pure BHZ system without altermagnetism ($J = 0$ regime): Floquet driving induces a transition from the trivial phase to the AFOTI-I with π- topological edge states, the conventional TI phase evolves into the AFOTI-II with π-hybridized edge states (the coexistence of 0- and π-ones). (c) Altermagnetism's symmetry-induced modulation mechanism: The arrows in Figure S2, linking $J = 0$ and $J \neq 0$ regimes, reveal that the AFOTI phases are transformed into the AHOTI ones upon introducing altermagnetism. This originates from the altermagnetism-induced breaking of TRS, which localizes 0D CMs.

S-3. FLOQUET MICROMOTION OPERATORS AND DYNAMICAL NESTED WILSON LOOP THEORY

A. Construction of Floquet Micromotion Operators

The dynamical analysis in this study is based on the rigorous construction of Floquet micromotion operators [1–4]

$$U_\varepsilon(\mathbf{k},t) = U(\mathbf{k},t)\,[U(\mathbf{k},T)]_\varepsilon^{-\frac{t}{T}}\,, \tag{S4}$$

where the core operator $[U(\mathbf{k},T)]_\varepsilon^{-\frac{t}{T}}$ characterizes *normal* Floquet dynamics. Its mathematical formulation is achieved through a quasi-energy branch cut operation on the time-evolution operator $U(\mathbf{k},T)$. Specifically, project the eigenvalues of $U(\mathbf{k},T)$ onto the ε-branch cut in the complex plane, and then apply a fractional time power $-\frac{t}{T}$ via exponentiation. The explicit expression is given by

$$[U(\mathbf{k},T)]_\varepsilon^{-\frac{t}{T}} = \sum_\alpha |E_\alpha(\mathbf{k})\rangle [e^{-iE_\alpha(\mathbf{k})}]_\varepsilon^{-\frac{t}{T}} \langle E_\alpha(\mathbf{k})|. \tag{S5}$$

When coupled with the time-evolution operator $U(\mathbf{k},t)$, $U_\varepsilon(\mathbf{k},t)$ exhibits periodicity invariance, whose dynamical evolution incorporates *anomalous* Floquet mode features. By tuning the branch cut parameter $\varepsilon \in \{0,\pi\}$, the topological responses of distinct quasi-energy gaps can be resolved,

$$[U(\mathbf{k},T)]_{\varepsilon=0}^{-\frac{t}{T}} = \sum_{\alpha=1}^{N/2} e^{-i(2\pi - E_\alpha(\mathbf{k}))\frac{t}{T}} \left|-E_\alpha(\mathbf{k})\right\rangle \left\langle -E_\alpha(\mathbf{k})\right| + \sum_{\alpha=N/2+1}^{N} e^{-iE_\alpha(\mathbf{k})\frac{t}{T}} \left|+E_\alpha(\mathbf{k})\right\rangle \left\langle +E_\alpha(\mathbf{k})\right|, \tag{S6}$$

$$[U(\mathbf{k},T)]_{\varepsilon=\pi}^{-\frac{t}{T}} = \sum_{\alpha=1}^{N/2} e^{iE_\alpha(\mathbf{k})\frac{t}{T}} \left|-E_\alpha(\mathbf{k})\right\rangle \left\langle -E_\alpha(\mathbf{k})\right| + \sum_{\alpha=N/2+1}^{N} e^{-iE_\alpha(\mathbf{k})\frac{t}{T}} \left|+E_\alpha(\mathbf{k})\right\rangle \left\langle +E_\alpha(\mathbf{k})\right|, \tag{S7}$$

where N is the total number of bands, and $|\pm E_\alpha(\mathbf{k})\rangle$ represent the positive/negative branches of the quasi-energy spectrum. Notably, the $\varepsilon = 0$ and π cuts correspond to edge-state characteristics near the 0- and π-gap, respectively.

B. Dynamical Nested Wilson Loop Theory

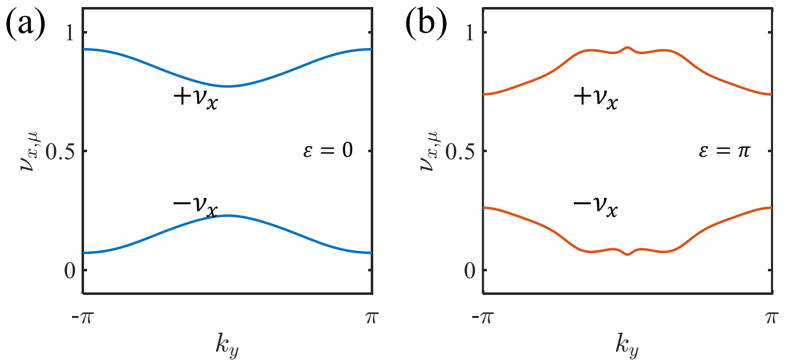

Figure S3: The first-order dynamic branches $\nu_{x,\mu}(k_y,t)$ with $T = 1$ and $t = T/2$ for the 0-gaps (a) and π-gaps (b), respectively.

Now, we introduce the dynamical nested Wilson loop method. In practical computations, since the analytical form of $U_\varepsilon(\mathbf{k},t)$ is difficult to obtain, we often employ discrete numerical approaches. First, the Brillouin zone is discretized to compute the first-order dynamical Wilson loop

$$\begin{aligned} Q_{x,\mathbf{k}}(t) &= \frac{\mathbb{I} + U_\varepsilon^\dagger(\mathbf{k}+\Delta_x\mathbf{e}_x,t)\,U_\varepsilon(\mathbf{k},t)}{2}, \\ W_{x,\mathbf{k}}(t) &= Q_{x,\mathbf{k}+(L_x-1)\Delta_x\mathbf{e}_x}(t)\cdots Q_{x,\mathbf{k}+\Delta_x\mathbf{e}_x}(t)Q_{x,\mathbf{k}}(t), \end{aligned} \tag{S8}$$

where $\mathbf{e}_x$ is the unit vector in the x-direction, and $\Delta_x = \frac{2\pi}{L_x}$ signifies the mesh size of the Brillouin zone. The eigenvalues and eigenvectors of $W_{x,\mathbf{k}}(t)$ fulfill

$$W_{x,\mathbf{k}}(t)|\nu_{x,\mu}(\mathbf{k},t)\rangle = e^{-2\pi i \nu_{x,\mu}(k_y,t)}|\nu_{x,\mu}(\mathbf{k},t)\rangle \tag{S9}$$

with $\nu_{x,\mu}(\mathbf{k},t)$ being the first-order dynamical branch, and the subscript μ indicating various branches. The static Wilson loop calculation typically employs wave functions of occupied states to form the $W_{x,\mathbf{k}}$ operator, thus the dimension of the static $W_{x,\mathbf{k}}$ is half the dimension of the Hamiltonian. However, in the dynamical scenario, the dimension of $W_{x,\mathbf{k}}(t)$ matches the Hamiltonian's dimension. Consequently, diagonalizing $W_{x,\mathbf{k}}(t)$ yields four ν_x values, where the branch set are defined as $\mu = 1,2 \in +\nu_x, \mu = 3,4 \in -\nu_x$ [see Figure S3(a) and (b)]. The system's mirror symmetry results in degeneracy between the two branches in each set. The presence of gaps for both $+\nu_x$ and $-\nu_x$ across the entire Brillouin zone with variations in k_y provides optimal conditions for the formation of the nested dynamic Wilson loop.

The construction of the second-order nested dynamic Wilson loop in the $\pm\nu_x$ subspaces is given by

$$W^{(\pm\nu_x)}_{y,\mathbf{k}}(t) = Q^{(\pm\nu_x)}_{y,\mathbf{k}+(L_y-1)\Delta_y\mathbf{e}_y}(t)\cdots Q^{(\pm\nu_x)}_{y,\mathbf{k}+\Delta_y\mathbf{e}_y}(t)Q^{(\pm\nu_x)}_{y,\mathbf{k}}(t) \tag{S10}$$

with the matrix elements

$$[Q^{(\nu_x)}_{y,\mathbf{k}}(t)]_{\mu_1\mu_2} = \sum_{m_1,m_2}[\nu_{x,\mu_1}(\mathbf{k}+\Delta_y\mathbf{e}_y,t)]^*_{m_1}[Q_{y,\mathbf{k}}(t)]_{m_1m_2}[\nu_{x,\mu_2}(\mathbf{k},t)]_{m_2} \tag{S11}$$

and $Q_{y,\mathbf{k}}(t) = \frac{\mathbb{I}+U^\dagger_\varepsilon(\mathbf{k}+\Delta_y\mathbf{e}_y,t)U_\varepsilon(\mathbf{k},t)}{2}$. Here, it is noted that $\Delta_y = \frac{2\pi}{L_y}$ and $\mathbf{e}_y$ represents the unit vector in the y-direction. Similarly, the eigenvalue equation for $W^{(\pm\nu_x)}_{y,\mathbf{k}}(t)$ is given by

$$W^{(\pm\nu_x)}_{y,\mathbf{k}}(t)|\nu^{\pm\nu_x}_{y,\mu'}(\mathbf{k},t)\rangle = e^{-2\pi i \nu^{\pm\nu_x}_{y,\mu'}(k_x,t)}|\nu^{\pm\nu_x}_{y,\mu'}(\mathbf{k},t)\rangle, \tag{S12}$$

where $\nu^{\pm\nu_x}_{y,\mu'}(\mathbf{k},t)$ signifies the second-order dynamical branch. Given that the dimension of $W^{(\pm\nu_x)}_{y,\mathbf{k}}(t)$ is constrained by the number of $\pm\nu_x$ subspaces, being half of the Hamiltonian, there exist two second-order dynamic branches. Then, the average dynamical polarization

$$\left\langle \nu^{(\pm\nu_x)}_{y,\mu'} \right\rangle(t) = \frac{1}{L_x}\sum_{k_x}\nu^{(\pm\nu_x)}_{y,\mu'}(k_x,t) \tag{S13}$$

reveals the topological features of the system.

S-4. TOPOLOGICAL PHASE CHARACTERIZATION VIA REAL-SPACE BULK QUADRUPOLE MOMENT

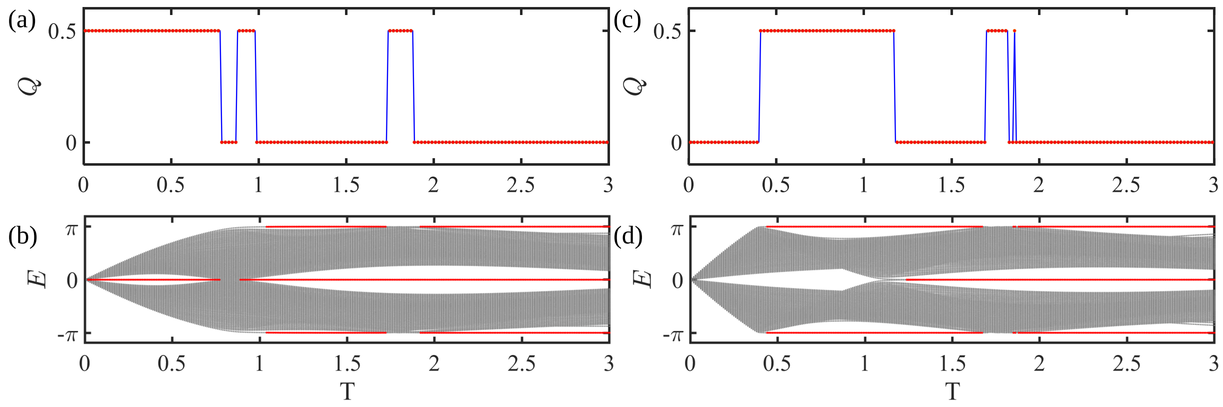

Figure S4: Evolution of the bulk quadrupole moment Q (a, c) and Floquet energy spectrum with the driving period T (b, d), where $m_0 = 1$ (initial HOTI phase) for (a) and (b), as well as $m_0 = 5$ (initial trivial phase) for (c) and (d). In (b) and (d), the red dots represent states at quasienergy 0 and π, respectively. Other parameters are $A_x = A_y = t_x = t_y = 2$, $J = 1$, $V_F = 10$, and $N_x \times N_y = 21 \times 21$.

When characterizing the higher-order topological phases in the system, we also introduce the real-space bulk quadrupole moment Q as a topological invariant. This quantity helps identify the presence of topologically protected corner modes (CMs) at either 0 or π quasienergy. The quadrupole moment is defined on the bases of occupied eigenstates in real space. For a Floquet system, these occupied states are derived from the eigenstates of the time-evolution operator $U(\mathbf{k}, T)$ at the end of one driving period, corresponding to all states below a specific quasienergy gap.

The computational procedure for Q is as follows. Consider a 2D lattice system of size $N_x \times N_y$, where each site has M internal degrees of freedom (e.g., spin and orbital). Let $\mathbf{u}$ be the matrix of occupied eigenstates, with dimension $(N_xN_yM) \times N_{\mathrm{occ}}$, where N_{occ} is the number of occupied states. We define a coordinate phase operator ϕ as [5–8]

$$\phi = \mathrm{diag}\left(\exp\left(i2\pi\hat{q}_{xy}\right)\right) \otimes I_M, \tag{S14}$$

where $\hat{q}_{xy} = \hat{x}\hat{y}/N_xN_y$ with $\hat{x}$ ($\hat{y}$) the position operator along the x (y) direction and N_x (N_y) the corresponding size of the 2D lattice system. Here, I_M denotes the $M \times M$ identity matrix and $\otimes$ represents the Kronecker product, ensuring the phase factor is applied to every internal degree of freedom.

Next, we construct a Wilson-loop-like operator

$$W = \mathbf{u}^\dagger \phi \mathbf{u}. \tag{S15}$$

The bulk quadrupole moment Q is then given by

$$Q = \frac{1}{2\pi}\mathrm{Im}\left[\ln\left(\det(W) \cdot \sqrt{\det(\phi^\dagger)}\right)\right]. \tag{S16}$$

Within this framework, a non-trivial higher-order topological phase (hosting either pure 0- or π-CMs) yields $Q = 0.5$ (mod 1), indicative of non-trivial quadrupole polarization. A trivial phase or a hybrid phase (AHOTI-II) results in $Q = 0$. It is important to note that near topological phase transitions, hybridization between corner modes and bulk states may cause significant oscillations and instability in Q, necessitating complementary spectral analysis for accurate phase identification.

Figure S4 displays the evolution of the bulk quadrupole moment Q and the energy spectrum as functions of the driving period T, for two values of the BHZ mass term m_0. Panels (a) and (b) correspond to $m_0 = 1$, while (c) and (d) correspond to $m_0 = 5$. When $Q = 0.5$, the spectrum clearly exhibits corner modes at either 0 or π energy, confirming the AHOTI-I or HOTI phase. When $Q = 0$, the system is in either the trivial or AHOTI-II phase, and the latter is distinguishable via the simultaneous presence of 0 and π modes in the spectrum. Around the topological phase transitions points (e.g., $T \approx 1(a), 1.86(b)$), Q exhibits notable fluctuations, reflecting enhanced sensitivity of the topological invariant due to hybridization between corner and bulk states.

In summary, the bulk quadrupole moment Q serves as a robust indicator for pure corner-mode phases, while spectral analysis remains essential for distinguishing hybrid topological phases. Through the combined use of Q and spectral analysis, this study clearly demonstrates the role of the Floquet driving period T in modulating the topological phase of the system.

S-5. EXPERIMENTAL DETECTION SCHEME AND PARAMETER ESTIMATION

A. Experimental Detection of Topological State and Altermagnetism

The topological phases revealed in this study can not only be theoretically characterized by topological invariants but also directly verified by the existence of edge states and CMs observed through advanced spectroscopic techniques. Scanning tunneling microscopy (STM), through spatially resolved measurements of the LDOS via differential conductance (dI/dV), enables the detection of edge/CMs. Specifically, when the probe is positioned at sample edges or corners and the bias voltage is tuned to energy windows corresponding to the 0- or π-quasi-gap, the dI/dV spectrum exhibits pronounced resonance peaks, serving as direct evidence of topologically protected edge or CMs [9–11]. Furthermore, the closure of the bulk gap during topological phase transitions can be dynamically monitored by STM. As the system approaches the critical point of a phase transition, the bulk electronic density of states near the Fermi level sharply increases. This results in a characteristic behavior of the bulk dI/dV curve, which evolve from a U-shape (open bulk gap) to a V-shape (gap closure) and then back to a U-shape (formation of a new topological phase), providing microscopic experimental criteria for phase transitions.

There are also mature schemes for the control and detection of altermagnetism in experiments. Through the switching effect of spin-orbit torques, it is possible to achieve 90° or 120° rotation of altermagnet Néel vectors [12, 13]. By combining crystal symmetry design and electrical control methods, even 180° reversal of the Néel vector orientation can be realized [14, 15]. For detecting the altermagnetic order parameter and its associated collective modes, angle-resolved photoemission spectroscopy

(ARPES) could be employed to resolve the momentum-dependent splitting in the electronic structure, and inelastic neutron scattering would allow direct measurement of spin-wave excitations in the bulk [16]. Surface-sensitive techniques such as magnetic circular dichroism in scanning tunneling microscopy could further probe the symmetry-breaking magnetic texture [17].

B. Experimental Parameters Estimation and Realization Pathways

To achieve controllable modulation of higher-order topological phases in Floquet-altermagnet/TI heterostructures, key experimental parameters must satisfy the following constraints (1) The Floquet driving frequency (ω) must be commensurate with the energy scale of the bulk gap (Δ), optimally spanning $1-50$ THz ($4-207$ meV) to align with conventional TIs. For instance, a driving frequency of $\omega \approx 24$ THz ($\hbar\omega \sim 100$ meV) effectively induces band reconstruction when $\Delta \sim 100$ meV. Representative materials include HgTe quantum wells ($\Delta \sim 10-30$ meV) [18] and Bi_2Se_3 thin films ($\Delta \sim 300$ meV) [19, 20], whose bandgap magnitudes satisfy this frequency-matching criterion. (2) The altermagnet strength (J) requires precise synergistic adjustment with Floquet driving parameters. First-principles calculations reveal that MnF_2 exhibits an altermagnetic exchange interaction of $J \sim 30$ meV [21], a value consistent with both the proposed driving frequency range and the bulk gap magnitudes of candidate TIs.

For experimental realization, we propose using HgTe/CdTe heterostructures or Bi_2Se_3 thin films as TI substrates, coupled with altermagnet layers such as RuO_2 (metallic phase) or MnF_2 (insulating phase). Periodic driving can be introduced via terahertz laser pulses (pulse width ~ps) or near-infrared optical rectification techniques. The band modulation process can be tracked via ARPES, and STM spatial-resolved spectroscopy can precisely resolve the localized properties of $0/\pi$-CMs. The above scheme provides a feasible technical pathway for translating theoretical predictions of higher-order topological phase transitions into experimental observations.

[1] A. K. Ghosh, T. Nag, and A. Saha, Time evolution of Majorana corner modes in a Floquet second-order topological superconductor, Phys. Rev. B **107**, 035419 (2023).

[2] B. Huang and W. V. Liu, Floquet Higher-Order Topological Insulators with Anomalous Dynamical Polarization, Phys. Rev. Lett. **124**, 216601 (2020).

[3] A. K. Ghosh, T. Nag, and A. Saha, Dynamical construction of quadrupolar and octupolar topological superconductors, Phys. Rev. B **105**, 155406 (2022).

[4] R.-X. Zhang and S. Das Sarma, Anomalous Floquet Chiral Topological Superconductivity in a Topological Insulator Sandwich Structure, Phys. Rev. Lett. **127**, 067001 (2021).

[5] C.-A. Li, B. Fu, Z.-A. Hu, J. Li, and S.-Q. Shen, Topological Phase Transitions in Disordered Electric Quadrupole Insulators, Phys. Rev. Lett. **125**, 166801 (2020).

[6] B. Kang, K. Shiozaki, and G. Y. Cho, Many-body order parameters for multipoles in solids, Phys. Rev. B **100**, 245134 (2019).

[7] W. A. Wheeler, L. K. Wagner, and T. L. Hughes, Many-body electric multipole operators in extended systems, Phys. Rev. B **100**, 245135 (2019).

[8] B. Roy, Antiunitary symmetry protected higher-order topological phases, Phys. Rev. Res. **1**, 032048 (2019).

[9] S. Kezilebieke, M. N. Huda, V. Vaňo, M. Aapro, S. C. Ganguli, O. J. Silveira, S. Głodzik, A. S. Foster, T. Ojanen, and P. Liljeroth, Topological superconductivity in a van der Waals heterostructure, Nature **588**, 424 (2020).

[10] L. Sang, Z. Li, G. Yang, M. Nadeem, L. Wang, Q. Xue, A. R. Hamilton, and X. Wang, Majorana zero modes in iron-based superconductors, Matter **5**, 1734 (2022).

[11] J.-X. Yin, S. H. Pan, and M. Zahid Hasan, Probing topological quantum matter with scanning tunnelling microscopy, Nat. Rev. Phys. **3**, 249 (2021).

[12] I. M. Miron, K. Garello, G. Gaudin, P.-J. Zermatten, M. V. Costache, S. Auffret, S. Bandiera, B. Rodmacq, A. Schuhl, and P. Gambardella, Perpendicular switching of a single ferromagnetic layer induced by in-plane current injection, Nature **476**, 189 (2011).

[13] L. Liu, C.-F. Pai, Y. Li, H. W. Tseng, D. C. Ralph, and R. A. Buhrman, Spin-Torque Switching with the Giant Spin Hall Effect of Tantalum, Science **336**, 555 (2012).

[14] L. Han, X. Fu, R. Peng, X. Cheng, J. Dai, L. Liu, Y. Li, Y. Zhang, W. Zhu, H. Bai, Y. Zhou, S. Liang, C. Chen, Q. Wang, X. Chen, L. Yang, Y. Zhang, C. Song, J. Liu, and F. Pan, Electrical 180° switching of Néel vector in spin-splitting antiferromagnet, Sci. Adv. **10**, eadn0479 (2024).

[15] Z. Zhou, X. Cheng, M. Hu, R. Chu, H. Bai, L. Han, J. Liu, F. Pan, and C. Song, Manipulation of the altermagnetic order in CrSb via crystal symmetry, Nature **638**, 645 (2025).

[16] J. Krempaský, L. Šmejkal, S. W. D′Souza, M. Hajlaoui, G. Springholz, K. Uhlířová, F. Alarab, P. C. Constantinou, V. Strocov, D. Usanov, W. R. Pudelko, R. González-Hernández, A. Birk Hellenes, Z. Jansa, H. Reichlová, Z. Šobáň, R. D. Gonzalez Betancourt, P. Wadley, J. Sinova, D. Kriegner, J. Minár, J. H. Dil, and T. Jungwirth, Altermagnetic lifting of Kramers spin degeneracy, Nature **626**, 517 (2024).

[17] O. Fedchenko, J. Minár, A. Akashdeep, S. W. D′Souza, D. Vasilyev, O. Tkach, L. Odenbreit, Q. Nguyen, D. Kutnyakhov, N. Wind, L. Wenthaus, M. Scholz, K. Rossnagel, M. Hoesch, M. Aeschlimann, B. Stadtmüller, M. Kläui, G. Schönhense, T. Jungwirth, A. B.

Hellenes, G. Jakob, L. Šmejkal, J. Sinova, and H.-J. Elmers, Observation of time-reversal symmetry breaking in the band structure of altermagnetic RuO_2, Sci. Adv. **10**, eadj4883 (2024).

[18] Z. D. Kvon, D. A. Kozlov, E. B. Olshanetsky, G. M. Gusev, N. N. Mikhailov, and S. A. Dvoretsky, Topological insulators based on HgTe, Phys.-Usp. **63**, 629 (2020).

[19] J. P. Heremans, R. J. Cava, and N. Samarth, Tetradymites as thermoelectrics and topological insulators, Nat. Rev. Mater. **2**, 17049 (2017).

[20] H. Zhang, C.-X. Liu, X.-L. Qi, X. Dai, Z. Fang, and S.-C. Zhang, Topological insulators in Bi_2Se_3, Bi_2Te_3 and Sb_2Te_3 with a single Dirac cone on the surface, Nat. Phys. **5**, 438 (2009).

[21] Y.-X. Li, Y. Liu, and C.-C. Liu, Creation and manipulation of higher-order topological states by altermagnets, Phys. Rev. B **109**, L201109 (2024).
